# Hemoptysis: unilateral pulmonary artery atresia? a case report

**DOI:** 10.1186/s13019-023-02255-9

**Published:** 2023-06-29

**Authors:** Aneel Maini, Sebastian Cousins, Tyler Holliday, Jessica Wang Memoli, Tajender Singh Vasu, Puja Gaur Khaitan

**Affiliations:** 1 Department of General Surgery, Georgetown University School of Medicine, MedStar Washington Hospital Center, Washington, DC USA; 2 Department of Pulmonology, MedStar Washington Hospital Center, Washington, DC USA; 3 Department of Pulmonary and Sleep Medicine, Virginia Heart, Falls Church, VA USA; 4 Department of General Surgery, Division of Thoracic Surgery, Georgetown University School of Medicine, MedStar Washington Hospital Center, 110 Irving Street, NW (G253), Washington, DC 20010 USA; 5 Department of Surgery, Division of Thoracic Surgery, Sheikh Shakhbout Medical City, Mayo Clinic, PO Box 11001, Abu Dhabi, UAE

**Keywords:** Congenital, Hemoptysis, Thoracic imaging, Unilateral pulmonary artery atresia

## Abstract

**Background:**

Asymptomatic, isolated cases of unilateral pulmonary artery atresia may present in adulthood with symptoms such as recurrent respiratory infections, dyspnea, hemoptysis, and pulmonary hypertension. Unlike previously reported patients that underwent surgical management for this pathology, the patient in this report had no chronic history of recurrent respiratory infections, dyspnea, or pulmonary hypertension, making a diagnosis prior to extensive imaging difficult.

**Case presentation:**

A 55-year-old male presented to our emergency department (ED) with a 3-day history of recurrent cough with 2–3 tablespoons of hemoptysis per episode, chills, and occasional wheezing. A computed tomography angiography (CTA) was performed, which identified a congenital absence of the left pulmonary artery and a right-sided aortic arch. Hypertrophied left intercostal and bronchial arteries were noted to be perfusing the left lung. V/Q scan confirmed a heterogeneous distribution of gas throughout both lung fields with 97% perfusion to the right lung, but no visualization of the left lung on the perfusion images. Given extensive collateral blood supply to the left lung, interventional radiology performed a GELFOAM® embolization of the hypertrophied left bronchial artery and two parasitized arteries from the left subclavian artery to minimize intra-operative blood loss. This was immediately followed by a left thoracotomy, pneumonectomy, intercostal muscle flap placement, and bronchoscopy. The procedure was 360 min long with a total of 1500 cc blood loss that was salvaged and re-infused. No additional blood products were administered. The patient remained intubated post-operatively and was transferred to the surgical intensive care unit. His postoperative course was complicated by troponin leak, rhabdomyolysis, delirium, and ileus, all of which resolved over time. He was discharged home on postoperative day seven and continues to do well one-year later.

**Conclusions:**

The patient in this report presented with several episodes of isolated hemoptysis but unlike previously reported cases of unilateral pulmonary artery atresia, he had no history of recurrent respiratory infections, dyspnea, or pulmonary hypertension. Although unilateral pulmonary artery atresia is a rare diagnosis, in patients with unexplained, isolated hemoptysis, further examination of the vasculature may be warranted, and surgical management may be beneficial in appropriate, symptomatic patients.

**Supplementary Information:**

The online version contains supplementary material available at 10.1186/s13019-023-02255-9.

## Background

Unilateral pulmonary artery atresia is characterized by a congenital absence of a pulmonary artery. It is often associated with congenital heart defects and thus frequently presents early and is typically diagnosed in infancy. However, asymptomatic, isolated cases of unilateral pulmonary artery atresia may present in adulthood with symptoms such as recurrent respiratory infections, dyspnea, hemoptysis, and pulmonary hypertension. Therefore, early detection and management is essential to prevent serious complications.

## Case presentation

We present a 55-year-old male with a history of hypertension, dyslipidemia, gastroesophageal reflux disease (GERD), and obstructive sleep apnea who presented to our emergency department (ED) with a 3-day history of recurrent cough with 2–3 tablespoons of hemoptysis per episode, chills, and occasional wheezing. He had presented to our ED several months prior with a similar complaint. During the initial admission, he reported one prior event of hemoptysis in his youth and a CTA was performed which revealed an atretic left pulmonary artery and right-sided aortic arch. However, his symptoms were believed to be related to a possible pneumonia and was started on intravenous antibiotics. A bronchoscopy was performed during this initial admission which demonstrated normal airway anatomy with active oozing from the left lower lobe that was managed with topical epinephrine; he was subsequently discharged. Two months later, another bronchoscopy was performed after a repeat episode of hemoptysis with no remarkable findings or endobronchial pathology.

**Fig. 1:**
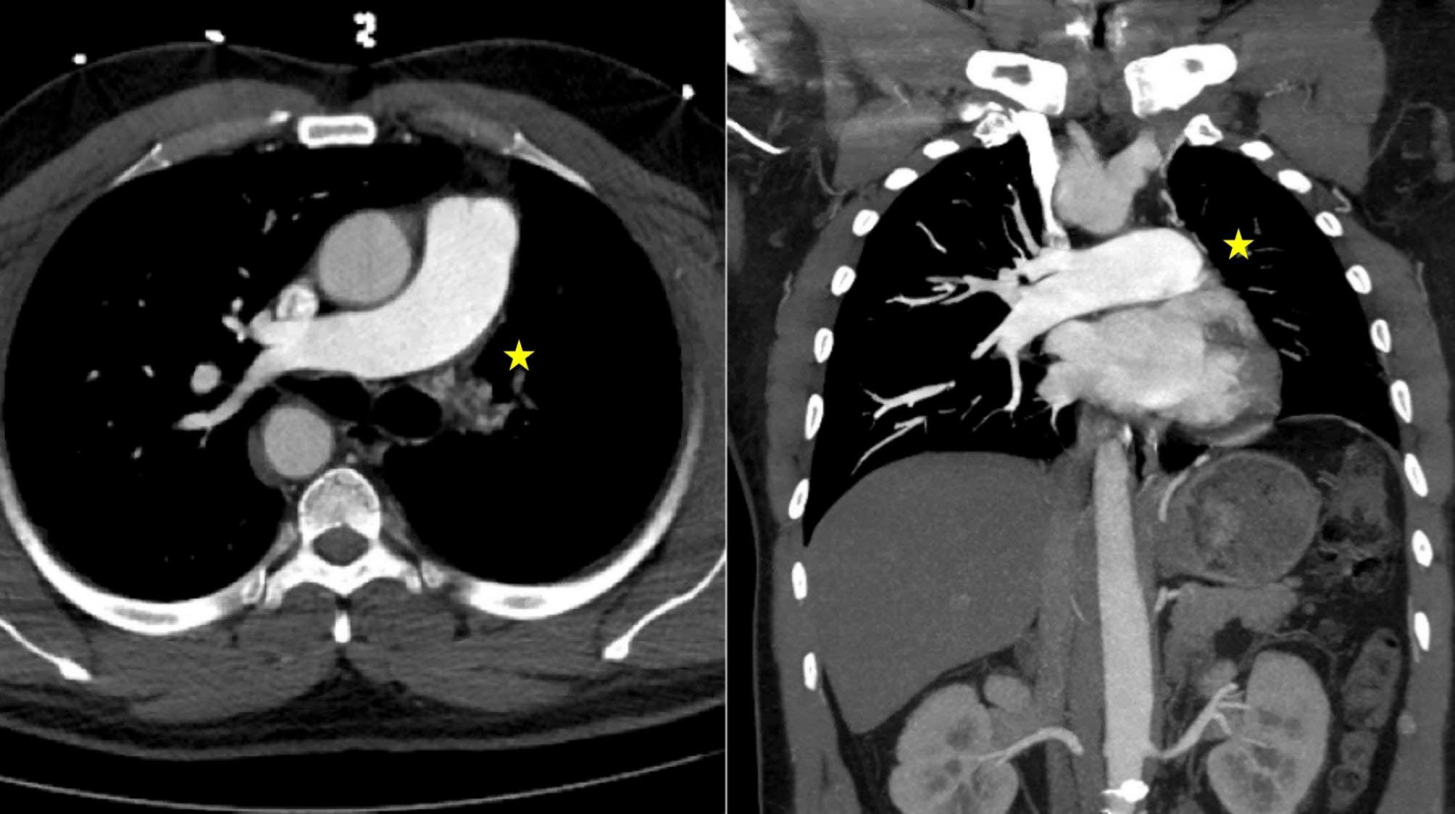
Computed tomography of an absent left pulmonary artery (identified with a yellow star)

**Fig. 2:**
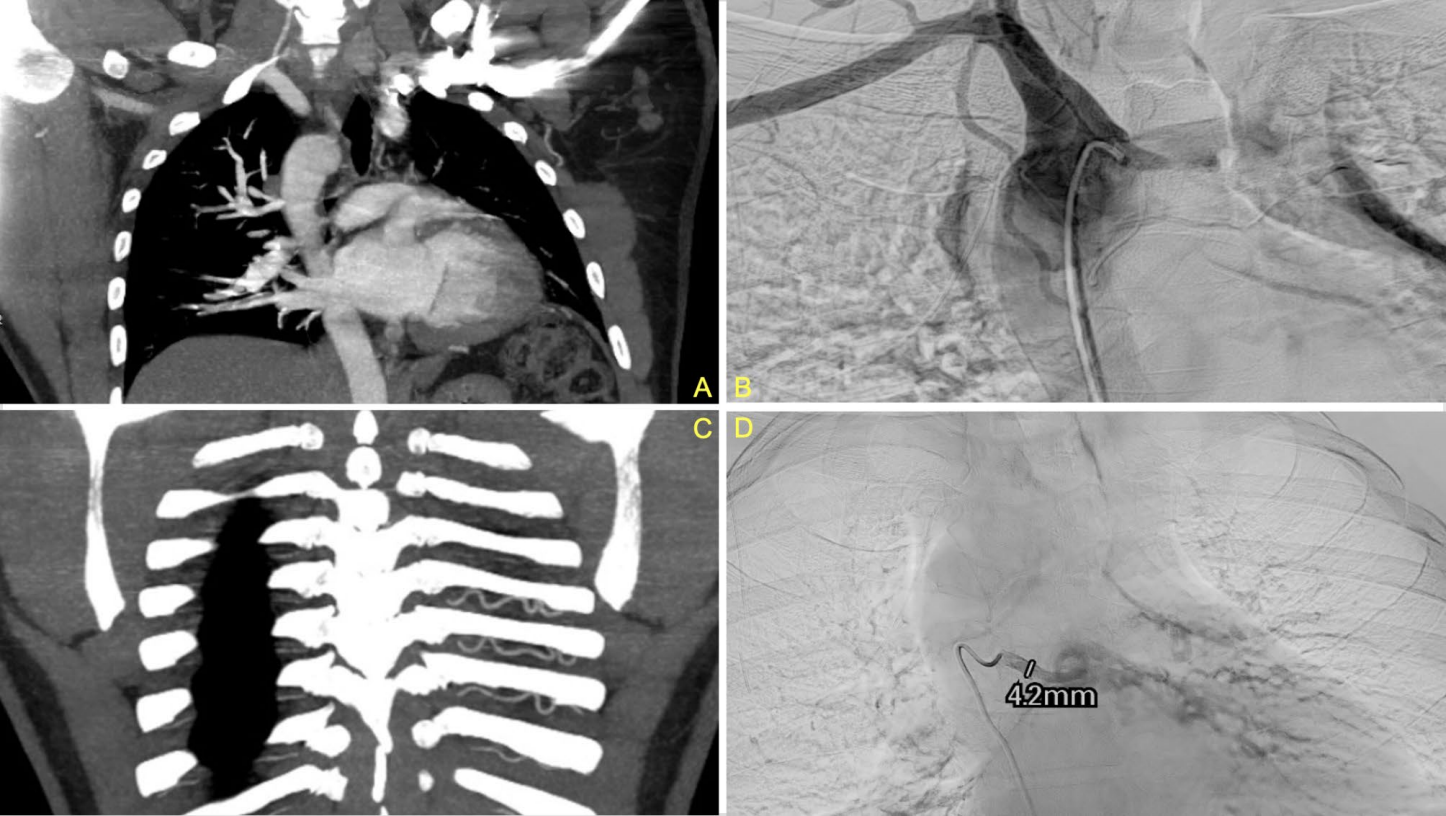
Computed tomography of right-sided aortic arch (**2a**). The patient demonstrated a variant arch vessel branching pattern with a left brachiocephalic artery as the first branch (not pictured), a right common carotid artery as the second branch, and a right subclavian artery as the third branch (**2b**) as well as hypertrophy of the intercostal and bronchial arteries (**2c** and **2d**)

**Fig. 3:**
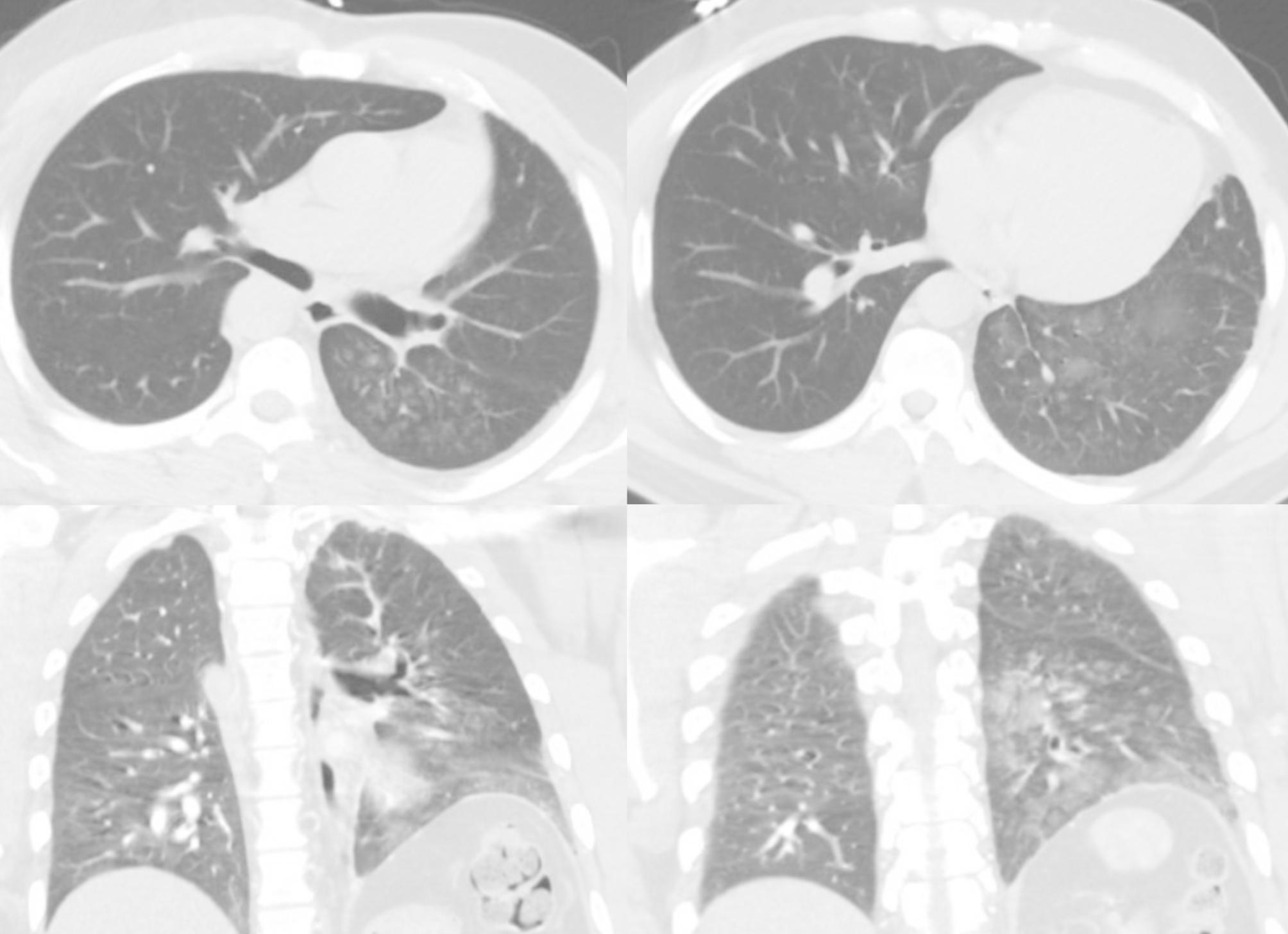
Pre-operative computed tomography of lung parenchyma in axial and coronal views

**Fig. 4:**
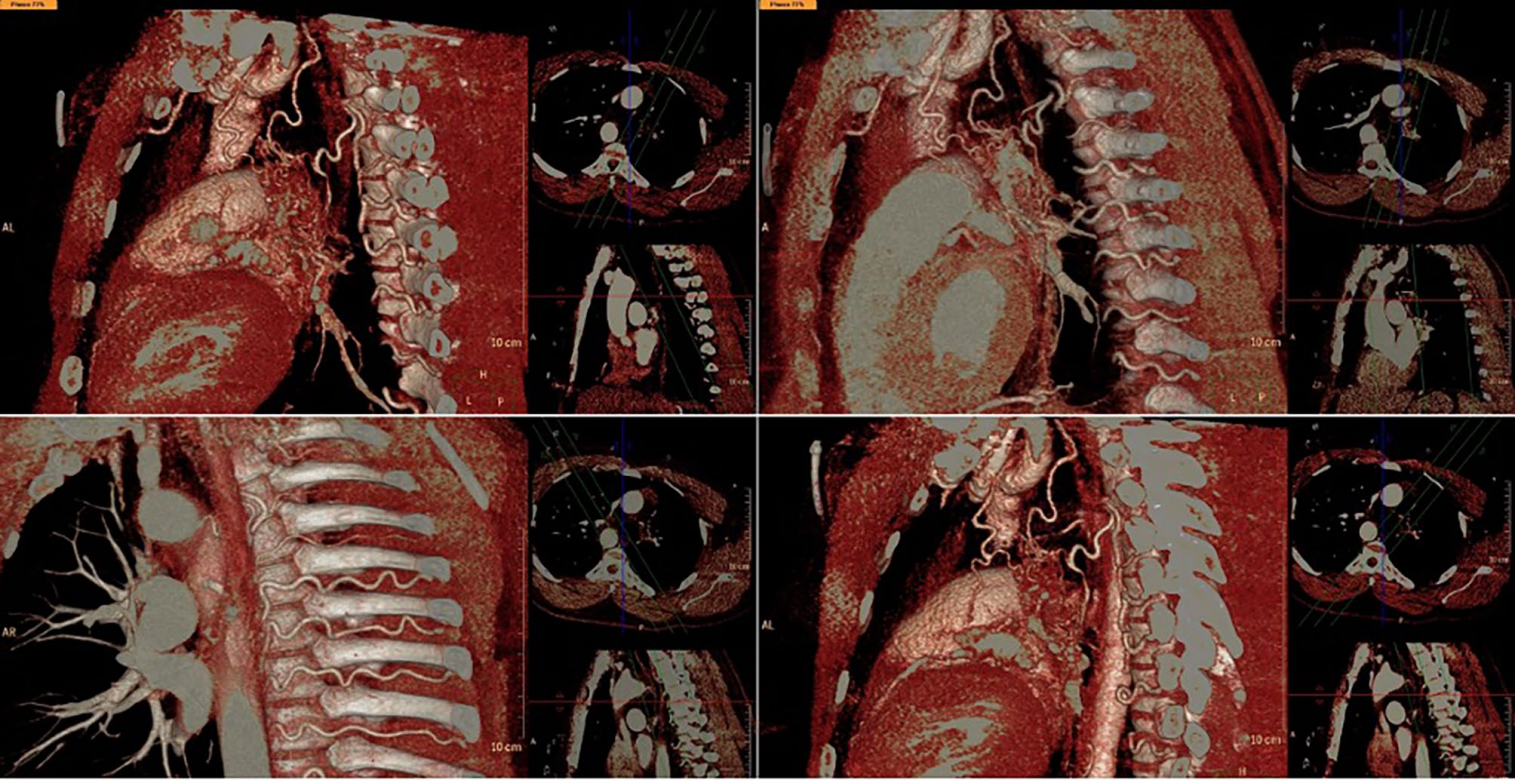
3-D computed tomography imaging reconstruction of the hypertrophied bronchial artery and intercostal arteries

Approximately nine months after the initial presentation, the patient began experiencing hemoptysis once again, prompting him to visit an urgent care center and was treated for what was once again believed to be a possible pneumonia. He began to experience increased frequency and larger-volume hemoptysis prompting him to present to our ED once again. A CTA was again performed which demonstrated a congenital absence of the left pulmonary artery (Fig. 1) as well as a right-sided aortic arch (Fig. 2a). Imaging also revealed an absent left superior pulmonary vein. Hypertrophied left intercostal and bronchial arteries (Fig. 2c and d) were noted to be perfusing the left lung (Fig. 3). This was confirmed on 3D reconstruction (Fig. 4). Once the diagnosis of unilateral pulmonary artery atresia was made, thoracic surgery was consulted, and the patient was worked up for a left pneumonectomy. Preoperative workup included an echocardiogram that confirmed preserved left and right ventricular function and no elevated pulmonary arterial pressures. Pulmonary function test showed no evidence of obstruction, moderate restriction, and a normal diffusion capacity to the right lung. V/Q scan confirmed a heterogeneous distribution of gas throughout both lung fields with 97% perfusion to the right lung, but no visualization of the left lung on the perfusion images.

**Fig. 5:**
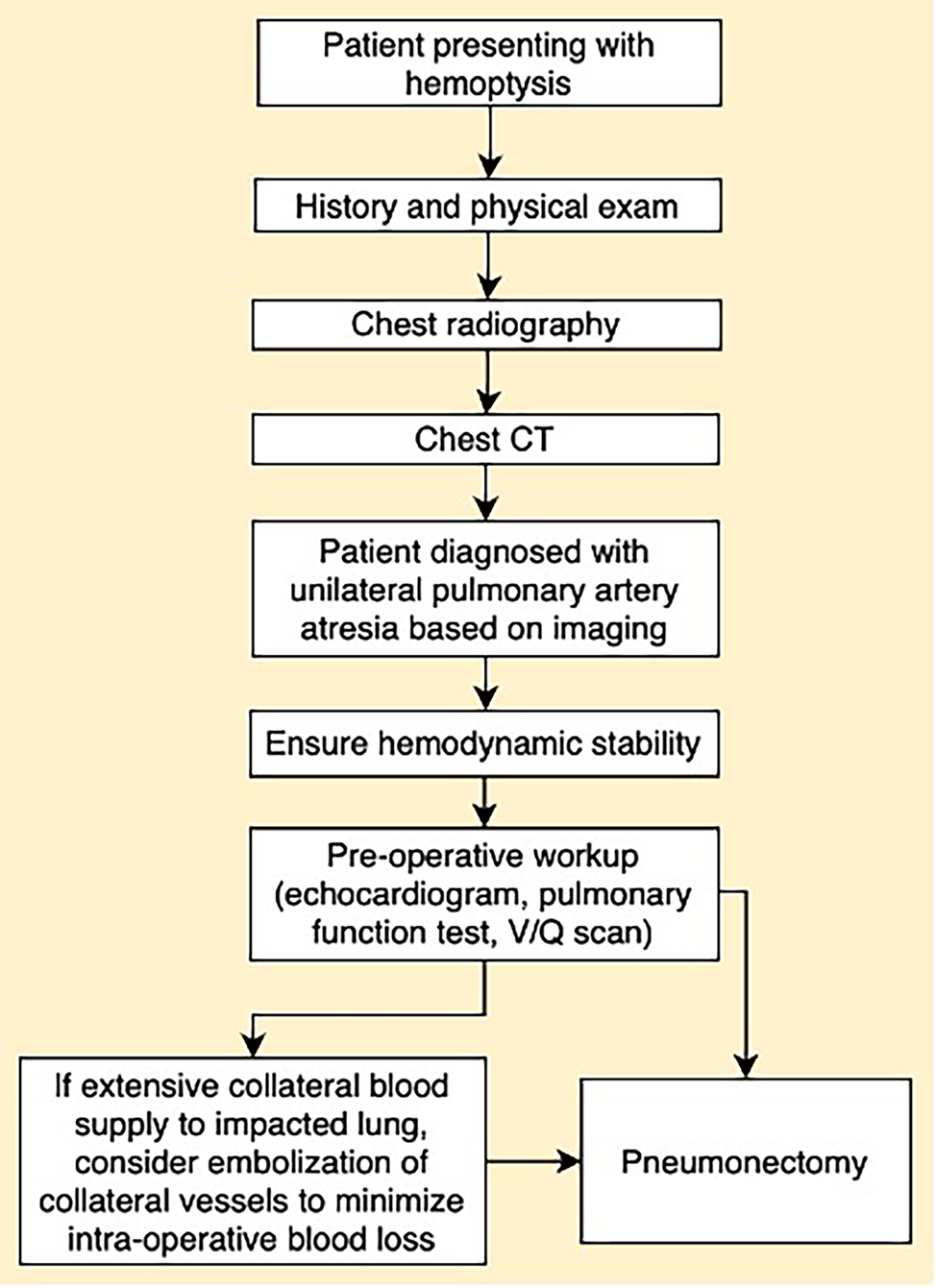
Flowchart detailing how our center diagnosed and managed this case of unilateral pulmonary artery atresia

Given extensive collateral blood supply to the left lung, interventional radiology performed a GELFOAM® (Pfizer, New York, NY) embolization of the hypertrophied left bronchial artery (Fig. 2d) and two parasitized arteries from the left subclavian artery to minimize intra-operative blood loss. This was immediately followed by a left thoracotomy, pneumonectomy, intercostal muscle flap placement, and bronchoscopy. Intra-operatively, multiple adhesions were carefully divided along the apex, diaphragm, and lateral chest wall where collateral circulation was divided with a combination of vascular staplers, vascular clips, ligation, and bipolar cautery. Multiple feeding vessels were noted coming off the internal thoracic, intercostals, and phrenic artery. An atretic inferior and a generous superior pulmonary vein were divided with vascular staplers. The bronchus was divided with a bronchial stapler and covered with an intercostal muscle flap. The lung parenchyma appeared boggy and thickened and adherent to the pleural planes and diaphragm; the fissure was fused and the hilum rather socked in both at the apex and at the inferior pulmonary ligament with an atretic inferior pulmonary vein, requiring meticulous dissection before division of critical structures. The procedure was 360 min long with a total of 1500 cc blood loss that was salvaged and re-infused. No additional blood products were administered. The patient remained intubated post-operatively and was transferred to the surgical intensive care unit. His postoperative course was complicated by troponin leak, rhabdomyolysis, delirium, and ileus, all of which resolved over time. He was discharged home on postoperative day 7 and continues to do well 1 year later. A flowchart detailing how our center diagnosed and managed this case of unilateral pulmonary artery atresia has been included in this report (Fig. 5).

## Discussion

Congenital absence of a pulmonary artery was first described by Frantzel O. Angeborener.

in 1868. It is thought to be caused by involution of the proximal sixth aortic arch on the affected side and persistence of the connection between the intrapulmonary artery and the distal sixth aortic arch, which eventually becomes the ductus arteriosus. These patients typically have a normal pulmonary trunk but have an absent branch of the pulmonary artery. The ductus arteriosus, or ligamentum arteriosum, has been shown to be ipsilateral to the absent pulmonary artery. It has been postulated that this is related to the persistent connection between the intrapulmonary pulmonary artery and the distal sixth aortic arch [1].

Due to the association with other cardiovascular abnormalities, most cases are diagnosed and surgically treated in the first year of life [2]. However, asymptomatic infants may not be diagnosed until adulthood, when the abnormality might be incidentally discovered on imaging or the patient may present with hemoptysis, recurrent respiratory infections, dyspnea, and/or pulmonary hypertension. Isolated absence of a pulmonary artery is estimated to occur in approximately 1 in 200,000 individuals [3]. Pulmonary hypertension is most frequently seen in cases where the patient has a concurrent cardiovascular shunt. Adult patients are also at risk of developing secondary bronchiectasis from recurrent infections [1]. In such patients that become symptomatic in adulthood, the diagnosis of unilateral pulmonary artery atresia is made via imaging due to the vague presenting symptoms and rarity of the pathology. As mentioned previously, the patient in this report underwent two bronchoscopies before the true diagnosis was made. Although there are no guidelines on the assessment or management of congenital absence of a pulmonary artery in adults, imaging allows other cardiovascular abnormalities that are associated with this pathology to be discovered. Besides the associated pathologic findings discussed below, other possible findings on CT include pleural thickening and parenchymal changes [4]. To better visualize the abnormal anatomy of the patient described in this report, a 3-D reconstruction was created (Video 1).

Congenital absence of a pulmonary artery can occur as an isolated condition or may be associated with other congenital heart defects such as tetralogy of Fallot, truncus arteriosus, right-sided aortic arch, septal defects, and/or patent ductus arteriosus. Patients with an absent left pulmonary artery are more frequently associated with congenital heart defects, and absence of the left pulmonary artery is more commonly seen in patients with tetralogy of Fallot and truncus arteriosus whereas absence of the right pulmonary artery is often an isolated finding but may be associated with a patent ductus arteriosus [1, 5]. Finally, approximately 50% of patients with an absent left pulmonary artery will have a right-sided aortic arch while those with an absent right pulmonary artery typically have a normal, left-sided aortic arch. Collateral pulmonary blood flow develops most commonly from the bronchial arteries (70%), phrenic arteries (50%), internal thoracic artery (40%), intercostal arteries (40%), subclavian or axillary artery (30%), directly from the aorta (10%), and/or the esophageal branches (10%) [6]. These collateral vessels eventually hypertrophy, dilate, and may rupture resulting in hemoptysis.

Surgical treatment options for adult patients presenting with congenital absence of a pulmonary artery include pneumonectomy, lobectomy, and ligation and embolization of arteriovenous malformations. Based on our review of the prior literature, there have only been a handful reports to date of pneumonectomy for symptomatic congenital absence of a pulmonary artery in an adult and merely three reports describing surgical treatment of a left sided absence.

The first case was a 35-year-old gentleman with a history of recurrent respiratory tract infections and exertional dyspnea who presented with hemoptysis and respiratory distress. CT scan demonstrated a left sided pulmonary artery interruption and a right-sided aortic arch. Embolization was performed, with initial cessation of the patient’s hemoptysis. However, recurrence of mild hemoptysis occurred within one week and the patient subsequently underwent repeat embolization followed by a left pneumonectomy. The authors reported no repeat hospitalizations or respiratory infections 18 months post-operatively [7]. Another report described a case involving a 70-year-old gentleman who presented with exertional dyspnea and productive cough but no hemoptysis and was found to have an absent left pulmonary artery with a right-sided aortic arch. Collateral arteries arising from the left internal mammary artery were identified. Embolization followed by pneumonectomy was performed and the patient made an uneventful recovery [8]. The third case was of a 52-year-old lady who had a known congenital absence of the left pulmonary artery and a right-sided aortic arch. Contrary to the patient in our report, this patient had a history of recurrent left-sided pulmonary infections. The patient eventually underwent a pneumonectomy with resolution of her recurrent infections [9].

Beyond the patient in our report remaining asymptomatic until the age of 55 and our management strategy, the described case was unique as the patient underwent multiple bronchoscopies with no clear etiology behind his hemoptysis until re-evaluation of his vasculature was performed. As mentioned previously, the initial bronchoscopy demonstrated active oozing from the left lower lobe which was managed with topical epinephrine. Two months later, another bronchoscopy was performed after a repeat episode of hemoptysis with no remarkable findings or endobronchial lesions.

## Conclusion

The patient in this report presented with several episodes of isolated hemoptysis, but unlike previously reported cases of surgically managed left sided pulmonary artery atresia, he had no history of recurrent respiratory infections, dyspnea, or pulmonary hypertension. Prior to diagnosis ultimately being made via CTA imaging, his presenting hemoptysis was attributed to an underlying pneumonia, prompting the use of intravenous antibiotics as well as two bronchoscopies. Based on prior literature, the decision was made to undergo embolization of the collateral vessels supplying the left lung to minimize intra-operative blood loss immediately followed by pneumonectomy. The patient continues to do well on follow-up 1 year post-operatively.

In summary, although unilateral pulmonary artery atresia in adults is a rare diagnosis with vague presenting symptoms, in patients with unexplained, isolated hemoptysis, further imaging of the vasculature may be warranted, and surgical management via embolization of the collateral vessels supplying the impacted lung followed by pneumonectomy can be performed safely and effectively with planned approach.

## Electronic supplementary material

Below is the link to the electronic supplementary material.

Supplementary Material 1: Video 1: 3-D computed tomography reconstruction demonstrating an absent left pulmonary artery and a right-sided aortic arch with a variant arch vessel branching pattern. Unfortunately, image artifacts limit the quality of the 3-D reconstruction and proper visualization of the left subclavian artery.

## Data Availability

Not applicable, case report.

## Abbreviations

CTA: Computed tomography angiography
